# Racial and Ethnic Diversity in SARS-CoV-2 Vaccine Clinical Trials Conducted in the United States

**DOI:** 10.3390/vaccines10020290

**Published:** 2022-02-14

**Authors:** Lana Khalil, Maranda Leary, Nadine Rouphael, Ighovwerha Ofotokun, Paulina A Rebolledo, Zanthia Wiley

**Affiliations:** 1 The Hope Clinic of the Emory Vaccine Center, Division of Infectious Diseases, Department of Medicine, Emory University School of Medicine, 500 Irvin Court Suite 200, Decatur, GA 30030, USA; lana.khalil@emory.edu (L.K.); maranda.leary@emory.edu (M.L.); preboll@emory.edu (P.A.R.); zwiley@emory.edu (Z.W.); 2 Division of Infectious Diseases, Department of Medicine, School of Medicine, Emory University, Atlanta, GA 30030, USA; iofotok@emory.edu

**Keywords:** racial disparities, ethnic disparities, clinical trials, COVID-19, H1N1

## Abstract

Evidence shows that White and non-Hispanic individuals are overrepresented in clinical trials. The development of new vaccines and drugs, however, necessitates that clinical research trials include representative participants, particularly in light of evidence showing that underrepresented minorities may have a different response to certain medications and vaccines. Racial and ethnic disparities among clinical trials are multilayered and complex, and this requires action. The results of this study indicate that significant racial and ethnic disparities consistently exist among the most recent early SARS-CoV-2 vaccine clinical trials as compared to the pandemic H1N1 vaccine clinical trials of 2009. New strategies, policies, training programs, and reforms are required to address these disparities among clinical trials.

## 1. Introduction

Coronavirus disease (COVID-19) has disproportionately impacted United States (U.S.) communities of color, particularly Black or African American, Hispanic or LatinX, and American Indian and Alaska Native communities [1]; thus, an adequate representation of communities of color in clinical trials, especially in COVID-19 vaccine clinical trials, is essential to reflect the population at the highest risk for COVID-19 acquisition and increased severity of disease.

Concerted research efforts have accelerated the licensure of several vaccines through rigorous clinical trials detailing tolerability, safety, immunogenicity, and efficacy of different vaccine candidates [2,3]. As of January 2022, there are three approved or authorized vaccines in the U.S.: two using mRNA-based technology (Pfizer BioNTech [BNT162b2] and Moderna [mRNA-1273]), and one using a non-replicating viral vector vaccine (Johnson and Johnson adenovirus Ad26), all encoding the spike protein. These vaccines received approval by February 2021, and two were fully approved in August 2021 (Pfizer BioNTech [BNT162b2]) and January 2022 (Moderna [mRNA-1273]). While many COVID-19 vaccine clinical trials were implemented and completed enrollment in record time allowing life-saving countermeasures, many clinical trials have not succeeded in adequately recruiting populations that reflect the U.S. demographics [4]. Historically, clinical trials have underreported race and ethnic subgroup analysis, especially in regard to Black race and Hispanic ethnicity [5]. This is particularly relevant to racially diversified societies, such as the U.S. one, because research has shown survival variances from diseases by race, even after controlling for differences in socioeconomic status and treatments [5]. For COVID-19, numerous studies showed associations of male sex, older age, Black or African American, American Indian, and Alaska Native races, Hispanic or LatinX ethnicity, and preexisting medical conditions with poor outcomes [4]. Well-designed clinical trials should evaluate vaccine candidates in populations most at risk for disease acquisition and severe disease [6]. Racially associated genetic variation can be an important determinant of an investigational product response [7]. Even with current efforts at tackling enrollment diversity in clinical trials, diversity remains low, especially in early-phase clinical trials [8,9]. Khubchandani et al. showed that the overall pooled prevalence rate of COVID-19 vaccination hesitancy from 13 different studies [n = 107,841 participants] was 41.6% for Black or African Americans and 30.2% for Hispanics or LatinX [10]. For both underrepresented racial and ethnic groups, there were many predictors of hesitancy, with the most common ones being the following: sociodemographic characteristics, medical mistrust, history of racial discrimination, and misinformation [11]. Perceptions of rapid development of vaccine candidates and politization of vaccine recommendations were listed as factors associated with COVID-19 vaccine hesitancy [10]. The underrepresentation of racial and ethnic minorities limits the generalizability of research findings and displays disparity in access to high-quality health care delivery. It also misses the opportunity to engage communities during the initial stages of vaccine development and help with establishing trust in the process, which can ultimately lead to a better understanding of the various stages of vaccine clinical trials through promotion of health education and ultimately continuous engagement in research. Although improving clinical trial diversity is a moral community goal, it should also be improved for scientific reasons. Multiple studies proved that there is a fundamental biological association with the social construct of race and emphasized the need to understand this complexity [5]. The lack of equitable representation will hinder progress into the era of precision medicine.

## 2. Materials and Methods

### 2.1. Study Design

We collected data from the main reported COVID-19 vaccine clinical trials conducted solely in the U.S.A. and compared them with data from a representative sample of early-phase clinical trials on vaccines against the prior 2009 pandemic H1N1 influenza virus. The terms “vaccine,” “vaccination,” “immunization”, and “clinical trial” were used to identify clinical trials that were registered in ClinicalTrials.gov, funded by the U.S. government or industries including (1) all age groups, (2) children only, adults only, or both children and adults, and (3) targeting vaccines against Severe Acute Respiratory Syndrome Coronavirus 2 (SARS-CoV-2) as well as against the pandemic H1N1 influenza virus. The results of the included clinical trials needed to be available on clinicaltrial.gov or PubMed by 30 September 2021. Clinical studies performed completely or in part outside the U.S. were excluded. Since human participants were not involved in this review of the literature, and only publicly available data were used, review and approval by an institutional review board or ethics committee were not required.

### 2.2. Data Collection

Demographic data on race and ethnicity were collected using the categories by the Office of Management and Budget Standards for the Classification of Federal Data on Race and Ethnicity [12]: White, Black or African American, Asian, American Indian or Alaska Native, Pacific Islander or Native American, as well as multiracial, in addition to Hispanic or LatinX or not. Clinical trials were categorized by clinical trial phase (e.g., early-phase clinical trials such as Phase 1, 2, or 1/2 and late-phase clinical trials or phase 3 efficacy clinical trials), number of participants, age range of the participants, type of vaccine platform tested, pathogen targeted (SARS-CoV2 or pandemic H1N1 influenza). For comparison of these demographic data, we used U.S. population data from 2011 (for the pandemic H1N1 influenza vaccine clinical trials) and 2018 (for the SARS-CoV-2 vaccine clinical trials), from the American Community Surveys [e.g., US census] [13,14].

## 3. Results

A total of eight COVID-19 clinical trials conducted solely in the U.S. were identified, and all met the inclusion criteria for the final analysis [15,16,17,18,19,20,21,22,23]. Of the clinical trials included, seven were phase 1/2, and one was phase 3 [15,16,17,18,19,20,21,22,23] (Table 1). Two studies were performed in the pediatric population [16], while seven studies included adult participants. The number of participants varied depending on the phase of the vaccine clinical trial; in particular, it varied between 40 elderly participants for the early-phase 1 clinical trial of the Moderna (mRNA 1273) vaccine to 30,351 participants for the phase 3 efficacy clinical trial testing the same vaccine product [18,21]. All clinical trials were multicenter studies, with the phase 3 efficacy study including 99 sites across the U.S. [21]. The studies tested different vaccine products, with all studies testing the novel mRNA-based vaccines, and one study testing a protein-adjuvanted vaccine candidate [20]. All studies were implemented solely in the U.S. in 2020. The characteristics of these COVID-19 vaccine studies are summarized in Table 1. A representative sample of two clinical trials was chosen for the pandemic H1N1 influenza vaccines. Both clinical trials tested the pandemic monovalent unadjuvanted H1N1 influenza vaccine candidates in adults. The clinical trials were multicenter studies and enrolled between 406 and 805 participants in the summer of 2009 using a well-established vaccine-manufacturing platform. The characteristics of the pandemic H1N1 vaccine studies are summarized in Table 2. For the vaccine clinical trials there is, typically, a screening and enrollment visit followed by in-person and telephone follow-up visits, in addition to the use of participants’ diaries to document the reactogenicity of vaccines. The participants were followed for many months, and blood samples as well as other clinical samples were obtained to assess vaccine safety, immunogenicity, and efficacy (for phase 3 vaccine clinical trials).

In the Pfizer BioNTech (BNT162b20) mRNA vaccine study, there was a preponderance of White race among all age group distributions, with 86% of the participants between the ages of 12 and 15 years, 83% between 16 and 25 years, and 89% being 18 years old and above. Black or African Americans represented only 3–9% of the participants, and Asians 1–8%. Similarly, in the Moderna (mRNA-1273) phase 1, 2, and 3 clinical trials, White participants were overrepresented, accounting for 89–98% of the enrollments for the phase 1/2 clinical trial and 79% for the phase 3 clinical trial. While in the Moderna phase 1/2 study Hispanic or LatinX participants were no more than 13% of the participants, in the phase 3 study they were 21%. In the Sanofi (VAT00001) study, White participants represented 87% of the enrollments, while Black or African Americans and Asians participants represented 6% and 7%, respectively. Compared with the 2009 pandemic H1N1 influenza clinical trials, White persons comprised 91% of the enrollments, while Black or African Americans and Asian persons comprised 5% and 1%, respectively. Hispanic or LatinX persons comprised no more than 4% of the participants in the 2009 pandemic H1N1 influenza vaccine clinical trials.

## 4. Discussion

Barriers to enrolling a diverse population of patients in clinical trials are multilayered and complex. We show that White participants were overrepresented, and Black or African American, American Indian or Alaska Native adults, and Hispanic or LatinX participants were underrepresented, especially in early-phase pandemic vaccine adult clinical trials including those regarding the current COVID-19 vaccine. This finding is similar to what was observed for early-phase clinical trials on vaccines against the 2009 pandemic H1N1 influenza virus. For the U.S.-based Phase 1/2 studies, Black or African American participant enrollment ranged from 1 to 9%, while for the phase 3 clinical trial, Black or African American participants were 10%. The Hispanic or LatinX ethnicity was also consistently underrepresented, but its enrollment showed a slight improvement since early clinical trials, as it ranged between 1 and 4% in the pandemic H1N1 influenza vaccine clinical trials and between 0 and 21% in the COVID-19 vaccine clinical trials (Table 1 and Table 2). This is likely explained by barriers at the level of the participants, sites, investigators, communities, and health care systems. Despite efforts successfully made at each level, multi-level interventions could have the greatest potential for success.

Barriers to accessing health services, poverty [24], health literacy challenges, and historic distrust contribute to the underrepresentation of people of color in both clinical and prevention trials. In 2019, the median U.S. household income varied widely and was the highest among Asians ($98,174), followed by Whites ($76,057), Hispanics or LatinX ($56,113), and Blacks or African Americans ($46,073) [25]. These income patterns may reflect the disparities in clinical trial participation noted in these trials, though information on subjects’ socioeconomic status and income was not collected in the COVID-19 and in the H1N1 pandemic influenza vaccine studies. Although household income and socioeconomic status may contribute to these clinical trial enrollment disparities, the grounds for participation in clinical trials are far more complex. Fewer clinical trials are available through resourced hospital systems where people of color are more likely to receive care, and people of color may be more likely to be ineligible to participate in clinical trials if they have certain medical comorbidities [26]. Other structural challenges include inability to reach the clinical trial site for individuals with limited flexibility in work schedules or childcare options, lack of paid time off from work, and reliance on public transportation where the risk of acquiring highly transmissible viruses (such as SARS-CoV2 or pandemic H1N1 influenza) is high without appropriate personal protective equipment and social distancing. One option to overcome these structural challenges is to offer research visits at the participants’ home or place of employment to increase the likelihood of enrollment as well as retention in a research study. In addition, some clinical trials require technology for monitoring or follow-up and may limit participation from individuals with limited usability of technological platforms or access to the internet, which may lead to increased barriers to participation among some people of color [27]. Some factors that impair enrollment, including the lack of access to the healthcare system and of health literacy, difficulties traveling to clinics, being away from work, and working from home, are often similar to those that result in delayed care, and hence generate a pervasive systemic issue [28,29]. Language barriers may also contribute to decreased clinical trial awareness and health literacy, even in Hispanic or LatinX persons who are bilingual.

With COVID-19 cases being tracked by race and ethnicity in the U.S., early data showed that the Black or African American and Hispanic or LatinX populations were disproportionately affected by the pandemic, with higher rates of hospitalization and mortality in comparison to the White population [1]. In the U.S., Black or African American and Hispanic or LatinX persons have higher rates of underlying medical conditions such as diabetes, hypertension, heart and kidney diseases, and obesity [30,31], which place them at risk for severe COVID-19 infection [32]. Although many comorbidities increase the risk for severe COVID-19 infection resulting in hospitalizations and death, a large contributing factor to these disproportionate effects on communities of color and to the comorbidities themselves, is structural racism, with its longstanding effects [33]. Discrimination in education, housing, healthcare, and employment each contribute to social determinants of health that place communities at greater risk for exposure to COVID-19 and increase the risk of morbidity and mortality [33].

For example, the matriarch of a multigenerational home (residing with her parents, partner, and children), who must leave her home during the peak of a pandemic surge to work as a public bus driver is at higher risk of contracting SARS-CoV-2 and bringing it home to her family (compared to members of a wealthy family who are able to socially distance at home for both work and school). Her family may also face housing insecurity and lack of healthcare insurance, which in some families may result in avoidance of healthcare due to cost. This lack of access to healthcare may result in late presentation during the course of COVID-19 illness, leading to decreased chances of recovery. The contribution of structural racism and social risk factors must be considered when working towards the inclusion of racial and ethnic minorities in clinical trials.

Given that morbidity and mortality affect underrepresented minorities for different health metrics, it is imperative to include them early on in preventative clinical trials. Such disparities can be due to deep-rooted issues such as structural inequities resulting in lower weakened socioeconomic status, persistently prevailing distrust toward health care institutions including, but not limited to, the infamous Tuskegee syphilis experiment, and religious beliefs that do not parallel the preventative and treatment practices [34]. In a pilot survey by Lynch et al., factors identified by African American physicians as possibly being the main disadvantages to enrollment in clinical trials included lack of patient awareness of studies [93%] and patient mistrust of the medical community [92%] [35]. However, even for other pandemics like H1N1 influenza, and despite high awareness of the pathogen and the well-established egg-based vaccine manufacturing, diverse populations were hesitant to enroll in early-phase clinical trials. In the pivotal phase 2 randomized clinical trial of monovalent pandemic 2009-H1N1 influenza vaccine, out of the 805 patients enrolled, 734 [91%] where White [23]. Lack of health literacy also results in vulnerability to myths and misinformation [28]. Additionally, persons with lower household income have greater barriers for learning about and enrolling in pandemic vaccines clinical trials and tend to live in neighborhoods with fewer opportunities to access healthcare or academic centers where the research is being conducted [11].

An early engagement of the community is essential when recruiting subjects from historically marginalized communities, due to both distrust from racism and lack of diversity among members of many research teams [36,37,38]. One example of community engagement programs includes the NIH Community Engagement Alliance Against COVID-19 Disparities which aims to increase community engagement, diversity, and inclusion in these communities [39]. Another program incorporates a Community Advisory Board (CAB), a diverse group of volunteers who provide investigators and their study teams with community input into study design and local procedures to ensure both diversity and feasibility of the study. Another limitation to the recruitment of persons residing in marginalized communities includes research payment-related policies that may require providing the subjects a social security number or the completion of a W-9 form [40]. Requests for either of these have the potential to discourage members of marginalized communities of color from participating in these research studies.

COVID-19 racial and ethnic disparities are not limited to the adults; studies have shown that in U.S. children, Black or African American or mixed race, and underlying medical comorbidities are strong predictors of hospitalization [41,42]. Adequate representation of these communities in COVID-19 vaccine clinical trials is essential. The COVID-19 pandemic has interrupted the education and social development of students, which in turn has impacted both parents and children [41,42]. Developing safe and efficacious vaccines for younger populations is essential to the safe resumption of in-school learning. In a multinational, placebo-controlled, clinical trial evaluating the safety, immunogenicity, and efficacy of the Pfizer BioNTech (BNT162b2) COVID-19 vaccine in 2260 adolescents 12 to 15 years of age, 83% were White, while Black or African American participants were only 9% [15]. Although children and adolescents generally experience milder COVID-19 than adults, severe illness still occurs in this population, and children play a crucial role in SARS-CoV-2 transmission [43,44]. Targeting the vaccination of children and adolescents of all races and ethnic backgrounds decreases cases among children and their families.

The National Institutes of Health [NIH] and the U.S. Food and Drug Administration [FDA] have recognized the importance of these described disparities and have taken steps to increase reporting and representation of minorities in research [12]. Possible solutions to aid in attracting more diverse participants include having a multiracial, multiethnic research team, including lead or principal investigators who are reflective of the participants, providing financial incentives for flexible research clinic hours, accessible locations, mobile units, telemedicine options, and sponsoring required recruitment plans for minorities. It is also important that institutions commit to ensuring that lead research investigators and trainees are representative of the communities of color that they serve and that they offer increased funding for health equity research [45]. NIH-funded vaccinology grants (only three nationwide, to our knowledge) or other training opportunities are crucial to increase knowledge in vaccinology and build a pipeline of diverse research scientists. Some proposed goals for achieving diversity, equity, and inclusion in clinical and translational research include, but are not limited to, funding community-oriented research and health equity research on par with biomedical research, ensuring that clinical trial enrollment reflects the diversity of the population with health conditions under study, and improving trust and understanding of science by shared investments with the community [45]. Some recommended plans of action from the Clinical and Translational Science Award National Consortium include strategies from the categories of Leadership, Training, Research, and Clinical Trials [45]. Example strategies in the category of clinical trials include partnering with trusted community groups in the design of data collection as well as clinical trial engagement procedures; hiring research staff from the communities where the research is being performed; training research teams on effective communication and cultural humility; ensuring that the research group includes researchers who have experience or expertise in health disparities and minority health [45]. In addition, improving the diversity of the health care and clinical trial workforces will further ensure culturally suitable training and education, create trust, and decrease language barriers (both verbally and via research consent forms and other materials) to enhance clinical trial participation and vaccination rates among minority groups. However, these stand-alone strategies will not be sufficient without a system that supports, and intentionally invests in, community engagement and training and retention of researchers and staff of color and without intentional, targeted, and culturally sensitive recruitment of subjects from marginalized communities.

The U.S. health care system must prioritize addressing health inequities and structural racism that contribute to these inequities. Methods to tackle structural racism are multipronged and include individual, organizational, community, and policy actions. One study by George et al. reported that racially based mistrust was not a negative predictor of study enrollment and that the race of the research staff did not influence the decision among Black or African American survey respondents [40]. The most significant predictors of likelihood of participation included acknowledgment of health status, employment, and healthcare satisfaction [40]. This emphasizes how, despite some personal or religious beliefs, most of the data indicate that the main predictor of lack of participation is deeply rooted in systemic issues. Hence, addressing stigmas and historically originated mistrust of the U.S. medical system with respect to research and clinical trials, education, physician communication, and compassion is key to changing individual perceptions [46,47]. Since physicians are usually the first to discuss referral to clinical trials, it is imperative that they are cognizant of the social challenges potential participants may face and show cultural humility as they strive to foster trust in research and bi-directional dialogue. For example, physician investigators and research teams must commit to learning about the history of racism in healthcare and undergo implicit bias training. Research organizations and teams can commit to workforce diversity and ensuring that their research staff reflect the population from which they recruit and that speak the languages of those they recruit and have informed consent forms in the native languages spoken by their participants. Research teams should have continued community engagement in the communities that they serve and ensure that community advisory boards are diverse and inclusive. In addition to that, the biomedical workforce training and curriculum needs to be restructured to address these issues, and the research staff should be trained in cultural competency and in how to function seamlessly irrespective of the target population. Policy changes are also vital in targeting systemic racism. Race- and ethnicity-related research and education reform are essential. By targeting individuals, organizations, community, and policies, the research community can continue to build trusting relationships with the communities that they serve, which will allow for increased interest, acceptance, and enrollment of underrepresented minorities in clinical trials. COVID-19 undoubtedly did highlight and did bring unprecedented nationwide attention to long-standing societal disparities that many minorities in the U.S.face. For this reason, it is imperative to address this challenge and future challenges both during this ongoing COVID-19 pandemic and long after its conclusion.

## 5. Conclusions

Despite efforts to eliminate racial and ethnic disparities, gaps in race reporting and incongruent representation continue to be seen in clinical trials. Black or African American and Hispanic or LatinX persons as well as other minorities are consistently underrepresented compared to the White and non-Hispanic U.S. population across clinical trials for treatment and prevention of various ailments. Reducing racial and ethnic disparities is a multidimensional task that extends beyond clinical trial accrual and publication, and there is a pressing need for dedicated disparity research, additional affirmative policies and, most importantly, social interventions to improve the representation of minority groups in research during pandemic and non-pandemic times.

## Figures and Tables

**Table 1:** Race and Ethnicity Representation in COVID-19 Vaccine Clinical Trials Conducted in the U.S.

Vaccine Tested	Pfizer BNT162b2	Pfizer BNT162b2	Pfizer BNT162b2	Moderna mRNA-1273	Moderna mRNA-1273	Moderna mRNA-1273	Sanofi VAT00001	Moderna mRNA-1273	[14]
Study	Frenck et al. [15]	Frenck et al. [15]	Walsh et al. [16]	Jackson et al. [17]	Anderson et al. [18]	Chu et al. [19]	Goepfert et al. [20]	Baden et al. [21]	
No. Participants	N = 2260	N = 1098	N = 105	N = 45	N = 40	N = 601	N = 179	N = 30,351	
Age Range (years)	12–15	16–25	≥18–<55 ≥65–<85	≥18–<55	≥18–<55 ≥56	≥18–<55 ≥55	18–49 ≥50	≥18–<65 ≥65	
Race, No. (%)									
White	1933 (86)	911 (83)	93 (89)	40 (89)	39 (98)	569 (95)	157 (87)	24,024 (79)	236,173,020 (72)
Black, African American	109 (5)	97 (9)	3 (3)	2 (4)	0 (0)	16(3)	10 (6)	3090 (10)	41,617,764 (13)
Asian	7 (<1)	8(1)	9 (8)	1 (2)	1 (2)	7(1)	12 (7)	1382 (5)	18,415,1985 (6)
American Indian, Alaska Native	143 (6)	43 (4)	0 (0)	1 (2)	0 (0)	3 (<1)	0 (0)	233 (1)	2,801,587 (<1)
Native Hawaiian, Pacific Islander	3 (<1)	4 (<1)	0 (0)	0 (0)	0 (0)	1 (<1)	0 (0)	67 (<1)	626,054 (<1)
Multiracial	52 (2)	31 (2)	0 (0)	0 (0)	0 (0)	4 (<1)	0 (0)	636 (2)	11,280,031 (3)
Unknown	13 (<1)	4 (<1)	0 (0)	1 (2)	0 (0)	0 (0)	0 (0)	637 (2)	
Ethnicity No. (%)									
Hispanic, LatinX	162 (11)	217 (20)	3 (3)	6 (13)	1 (2)	0 (0)	27 (15)	6235 (21)	59,763,631 (18)

**Table 2:** Race and Ethnicity Representation in Selected pandemic H1N1 influenza Phase 2 Vaccine Clinical Trials.

Vaccine Tested	Sanofi-Pasteur UD12415	IIV3	2011 Consensus [13]
Study	Chen et al. [22]	Frey et al. [23]	
No. Participants	N = 406	N = 805	
Age Range (years)	18–64 ≥65	18–64 ≥64	
Race, No. (%)			
White	369 (91)	734 (91)	227,167,013 (74)
Black, African American	20 (5)	44 (5)	38,395,857 (13)
Asian	6 (1)	12 (1)	14,497,185 (5)
American Indian, Alaska Native	0 (0)	201 (<1)	2,502,653 (<1)
Native Hawaiian/Pacific Islander	0 (0)	1 (0)	500,592 (<1)
Multiracial	11 (3)	8 (1)	1,846,491 (<1)
Unknown	0 (0)	6 (1)	
Ethnicity, No. (%)			
Hispanic/LatinX	6 (<1)	32 (4)	49,215,563 (16)

IIV: inactivated influenza vaccine.

## Data Availability

Not applicable.
